# Hombre de 45 años con neumonía grave, infección diseminada por citomegalovirus y agammaglobulinemia

**DOI:** 10.7705/biomedica.7524

**Published:** 2024-12-23

**Authors:** Mónica Fernandes-Pineda, Andrés F. Zea-Vera

**Affiliations:** 1 Departamento de Medicina Interna, Universidad del Valle, Cali, Colombia Universidad del Valle Universidad del Valle Cali Colombia; 2 Departamento de Microbiología, Facultad de Salud, Universidad del Valle, Cali, Colombia Universidad del Valle Universidad del Valle Cali Colombia; 3 Genetic Immunotherapy Section, Laboratory of Clinical Immunology and Microbiology, Division of Intramural Research, National Institute of Allergy and Infectious Diseases, National Institutes of Health, Bethesda, MD, USA Division of Intramural Research, National Institute of Allergy and Infectious Disease USA

**Keywords:** agammaglobulinemia, citomegalovirus, neumonía, adulto, Agammaglobulinemia, cytomegalovirus, pneumonia, adult

## Abstract

Se trata de un paciente de sexo masculino de 45 años con tos persistente de cuatro meses de duración, acompañada de fiebre y una significativa pérdida de peso.

En la tomografía de tórax, se observó una neumonía idiopática y los estudios posteriores identificaron una carga viral positiva para citomegalovirus (CMV) en el lavado broncoalveolar. La biopsia transbronquial confirmó inclusiones basófilas intranucleares indicativas de la infección por CMV. Los valores de las inmunoglobulinas fueron: IgA < 0,13, IgG < 3 e IgM < 0,25 g/L. La biopsia de médula ósea reveló un aumento del 80 % en el número de células, sin alteraciones morfológicas. Se indicaron estudios complementarios para la agammaglobulinemia.

## Abstract (en)

A 45-year-old male patient presented with a persistent cough lasting four months, accompanied by fever and significant weight loss.

A chest computed tomography revealed cryptogenic pneumonia, and subsequent investigations identified a positive cytomegalovirus (CMV) viral load in bronchoalveolar lavage. A transbronchial biopsy confirmed intranuclear basophilic inclusions indicative of CMV infection. Additionally, blood tests returned positive results for CMV. Immunoglobulin levels revealed IgA < 0.13, IgG < 3, IgM < 0.25 (g/L). Bone marrow biopsy indicated an 80% hypercellularity without morphological alterations. Additional studies for agammaglobulinemia were asked for.

Se trata de un hombre de 45 años, comerciante, originario de Cali, que consultó a un centro de nivel IV de atención en mayo del 2022 por tos persistente de cuatro meses de evolución, acompañada de fiebre y significativa pérdida de peso (8 kg). No manifestó ningún antecedente médico relevante, excepto por una infección respiratoria por SARS-CoV-2 no complicada dos meses antes.

En el examen físico inicial, presentaba palidez e hipotensión con disminución de los ruidos respiratorios en ambos campos pulmonares. En la radiografía de tórax del ingreso, se observaron infiltrados alveolares predominantemente en el campo basal del pulmón derecho (figura 1A). Se diagnosticó una neumonía grave y se inició tratamiento antibiótico con piperacilina-tazobactam y vancomicina.

**Figura 1:**
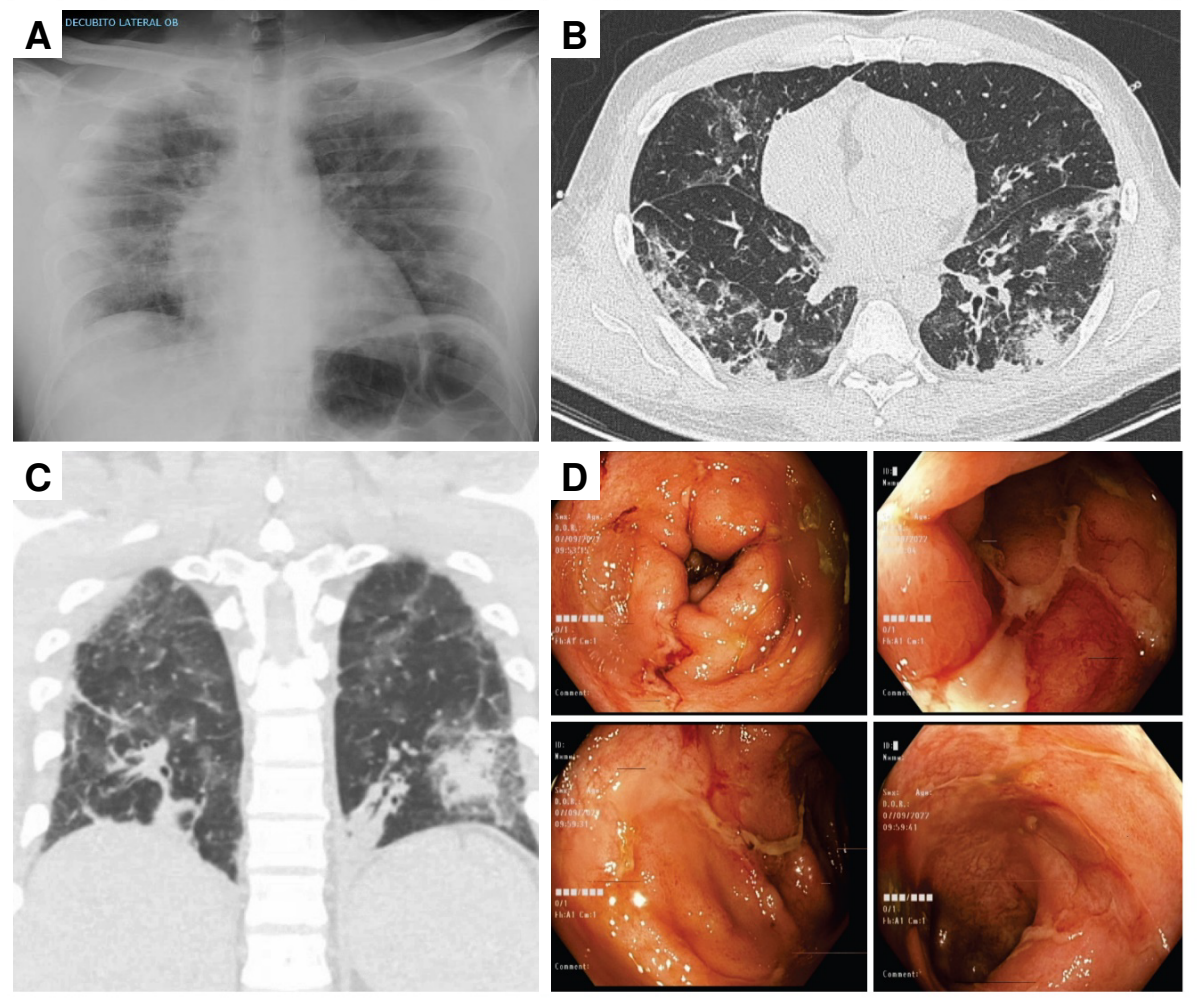
A. Radiografía de tórax: se observan infiltrados alveolares en el campo pulmonar derecho, con predominio basal. B. Tomografía computarizada de tórax, corte coronal: se aprecian múltiples focos bilaterales mixtos de opacidad periférica. C. Tomografía computarizada de tórax, corte transversal: se observa neumonía de carácter idiopático D. Colonoscopia total: en el segmento ascendente y el transverso se observan ulceraciones elongadas y de bordes definidos.

La tomografía computarizada de tórax evidenció una neumonía idiopática (figuras 1, B y C); en los laboratorios de ingreso, se reportó: procalcitonina elevada, 22,51 ng/ml (normal <0,5); sin leucocitosis (4.610 células/μl) con neutrófilos (2.480 células/μl), linfocitos (1.520 células/μl) y monocitos (560 células/μl) normales. El panel respiratorio (Filmarray™) fue negativo en el hisopado nasofaríngeo. En los estudios infecciosos complementarios del lavado broncoalveolar por fibrobroncoscopia, se encontró una carga viral positiva para citomegalovirus (CMV) de 995.000 copias/ml; el resultado fue negativo para bacterias, tuberculosis, hongos y para *Pneumocystis jirovecii*.

La biopsia transbronquial confirmó la presencia de inclusiones basófilas intranucleares, indicativas de infección activa por CMV, con PCR positiva en el tejido pulmonar para *P. jiroveccii*.

Durante la hospitalización, presentó múltiples episodios de diarrea, por lo cual se ordenó una colonoscopia en la se encontraron úlceras en el colon (figura 1D). La biopsia de colon y la inmunohistoquímica demostraron colitis crónica y ulceración, reportadas como negativas para CMV. La carga viral para CMV en sangre fue positiva, con 890 copias/ml. Por la infección activa diseminada por CMV se formuló ganciclovir, el cual recibió durante 21 días.

Los estudios para HIV (ELISA de cuarta generación y carga viral) fueron negativos; asimismo, se descartaron otras infecciones crónicas, como aquellas por HTLV, HCV o HBV.

Por tratarse de un paciente negativo para HIV y con una infección oportunista, se realizaron otros estudios para descartar neoplasias malignas hematológicas o tumores sólidos que explicaran su estado de inmunosupresión y el desarrollo de la infección grave por CMV. La biopsia de médula ósea mostró un aumento del 80 % en el número de células, con preservación de las tres líneas celulares y sin alteraciones morfológicas.

La citometría de flujo de médula ósea reveló asincronismo en la expresión de antígenos CD13 y CD10 en los granulocitos y neutrófilos. Los linfocitos B totales representaban el 0,35 % de los leucocitos totales (extremadamente bajo), con ausencia completa de linfocitos B maduros y muy escasas células plasmáticas.

En la PET-TC de cabeza y cuello, no se encontraron cambios metabólicos; en el tórax, había infiltrados bilaterales de predominio en el pulmón derecho, que se interpretaron como secundarios a la neumonía, mientras que el componente ganglionar mediastinal no evidenciaba cambios infecciosos. El hígado y el bazo no presentaban alteraciones aparentes.

Teniendo en cuenta el compromiso inmunológico del paciente, se consideró la posibilidad de un error innato de la inmunidad. La evaluación de la respuesta inmunitaria humoral mostró agammaglobulinemia en la electroforesis de proteínas (figura 2) y ausencia completa de inmunoglobulinas séricas, dada por: IgA < 0,13 g/L (0,7 - 4,9), IgG < 3 g/L (7 - 16) e IgM < 0,25 g/L (<0.25) ]. El recuento total de linfocitos T estaba discretamente aumentado, así, CD3+, 3.424 células/μl (849 - 1.963), con linfocitos T CD4+ normales, 1.115 células/μl (477 - 1.140) (31,9 % de los linfocitos T), pero la relación CD4/CD8 se encontraba invertida (0,48). Los linfocitos B y las células NK no se cuantificaron en esa oportunidad.

**Figura 2:**
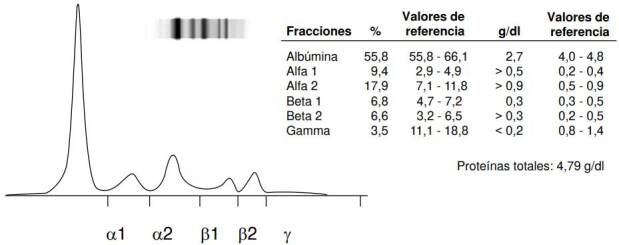
Electroforesis capilar de proteínas en suero con disminución casi completa de la región gamma (γ), indicativa de agammaglobulinemia. El eje de las abscisas representa el tiempo de migración de las proteínas según su carga y tamaño

Ante el hallazgo de hipogammaglobulinemia grave en el rango de agammaglobulinemia, sumado a la neumonía grave y al compromiso multisistémico por CMV, se consideró que el paciente cursaba con una inmunodeficiencia primaria o error innato de la inmunidad de tipo deficiencia predominantemente de anticuerpos. La ausencia de inmunoglobulinas séricas fue confirmada con una segunda muestra, pero, dada la premura y la gravedad del cuadro clínico, no se realizó evaluación funcional de la reacción inmunitaria humoral y se inició la administración de inmunoglobulinas por vía intravenosa cada 28 días, a una dosis de 400 mg/kg.

El paciente ha recibido de forma ininterrumpida la sustitución con inmunoglobulinas, a pesar de lo cual ha presentado dos episodios infecciosos respiratorios que requirieron tratamiento antibiótico.

El paciente fue valorado por el grupo de inmunología clínica de adultos, que consideró adelantar estudios de extensión y ordenó una prueba diagnóstica.

**Preguntas**

1. ¿Está de acuerdo con el diagnóstico inicial? ¿Cuáles serían los diagnósticos diferenciales?

2. ¿Qué estudios complementarios se solicitarían para este paciente?

